# Different Levels of Autophagy Activity in Mesenchymal Stem Cells Are Involved in the Progression of Idiopathic Pulmonary Fibrosis

**DOI:** 10.1155/2024/3429565

**Published:** 2024-02-15

**Authors:** Hongxia Tao, Qin Lv, Jing Zhang, Lijuan Chen, Yang Yang, Wei Sun

**Affiliations:** ^1^School of Medical and Life Sciences, Chengdu University of Traditional Chinese Medicine, Chengdu 611137, China; ^2^Department of Respiratory and Critical Medicine, Sichuan Provincial People's Hospital, Sichuan Academy of Medical Sciences, Chengdu, Sichuan, China; ^3^Medical College, University of Electronic Science and Technology, Chengdu, China

## Abstract

Idiopathic pulmonary fibrosis (IPF) is an age-related lung interstitial disease that occurs predominantly in people over 65 years of age and for which there is a lack of effective therapeutic agents. It has demonstrated that mesenchymal stem cells (MSCs) including alveolar epithelial cells (AECs) can perform repair functions. However, MSCs lose their repair functions due to their distinctive aging characteristics, eventually leading to the progression of IPF. Recent breakthroughs have revealed that the degree of autophagic activity influences the renewal and aging of MSCs and determines the prognosis of IPF. Autophagy is a lysosome-dependent pathway that mediates the degradation and recycling of intracellular material and is an efficient way to renew the nonnuclear (cytoplasmic) part of eukaryotic cells, which is essential for maintaining cellular homeostasis and is a potential target for regulating MSCs function. Therefore, this review focuses on the changes in autophagic activity of MSCs, clarifies the relationship between autophagy and health status of MSCs and the effect of autophagic activity on MSCs senescence and IPF, providing a theoretical basis for promoting the clinical application of MSCs.

## 1. Introduction

Idiopathic pulmonary fibrosis (IPF) is a chronic, progressive, irreversible, and fatal lung disease marked by lung scarring, with an average life expectancy of 3–5 years after diagnosis [1–4]. IPF primarily affects middle-aged and older adults; the prevalence of IPF increases with age among the numerous countries studied, with a high rate over 65 years [5]. The pathogenesis of IPF hinges on sustained or repetitive lung epithelial injury, which triggers the activation of fibroblasts and subsequent myofibroblast differentiation [6]. Two new approved therapies by the FDA, namely, pirfenidone and nintedanib, exhibit modest effectiveness in mitigating the decline in lung function over a 1-year follow-up period [7–10]. Nonetheless, these groundbreaking antifibrotic therapies are still in their nascent stages and are not frequently recommended for patients with a milder or stabilized course of the disease, primarily owing to the substantial incidence of side effects [10, 11]. Lung cancer frequently arises as a complication of IPF, with one-fifth experiencing acute exacerbations after treatment [12].

Cellular therapy for pulmonary fibrosis (PF) encompasses the application of mesenchymal stem cells (MSCs) [13]. MSCs are multipotent cells with the ability to differentiate into diverse cell types and bestow immunomodulatory, antiproliferative, and anti-inflammatory effects [14]. However, a multitude of internal and external factors have prompted alterations in the health status of MSCs, thus influencing their capacity to effectively facilitate the repair and regeneration of damaged lung tissue as therapeutic cells [15]. The regulation of autophagy within MSCs stands as a potential mechanism that could influence the properties of MSCs and potentially impact their regenerative and therapeutic potential [16]. Autophagy serves as the principal cellular process for breaking down and recycling intracellular proteins and organelles in various physiological and pathological contexts [17]. Impairment of autophagy fails to efficiently rectify malfunctioning organelles and eliminate detrimental metabolites within MSCs, ultimately resulting in the senescence of MSCs [18]. Excessive autophagy will lead to apoptosis of MSCs, affect the renewal ability of MSCs, and ultimately lead to the inability of MSCs to repair damaged lung tissue, accelerating the occurrence of IPF [19]. Therefore, the change of autophagy activity is closely related to the health status of MSCs.

In recent years, more and more researches have been committed to investigating the regulative network of autophagy in IPF [20, 21]. Autophagy is like a double-edged sword, indicating that autophagy activity may be a significant driving factor for IPF development [22]. Basal autophagy activity maintains pulmonary homeostasis in a cellular protective manner; it can selectively degrade potentially detrimental cytoplasmic substances, uneliminated proteins, and some unfavorable microorganisms, such as damaged organelles, viruses, protists, and bacteria [23]. In this review, this paper provides a focused review of the aging characteristics and functional changes of MSCs in IPF, as well as the mechanisms of autophagic activity affecting the health status of MSCs, to promote a more comprehensive application of MSCs in regenerative medicine.

## 2. The Emerging Role of Autophagy in IPF

### 2.1. The Biological Function of Autophagy

Autophagy represents the predominant cellular mechanism not only responsible for a bulk recycling system but also for targeting specific organelles, protein complexes, protein aggregates, and invading pathogens for catabolism [17]. According to the mechanism used to deliver cargo to the lysosome, autophagy can be classified as microautophagy, chaperone-mediated autophagy, and macroautophagy (MA) [24].

The mammalian target of rapamycin (mTOR) kinase is a conserved protein kinase involved in a multitude of cellular processes including nutrient sensing, cell growth, and autophagy, which is a signaling control point downstream of growth factor receptor signaling, hypoxia, ATP levels, and insulin signaling [25, 26]. mTOR kinase is a downstream effector of the PI3K/Akt pathway, signaling in the presence of nutrients and promoting cellular growth by stimulating the expression of ribosomal proteins and enhancing protein translation [27]. Crucially, mTOR also functions to suppress autophagy in these growth-favorable circumstances [28]. The activity of mTOR kinase is inhibited by signals that detect nutrient deficiency, such as hypoxia [29]. Upstream of mTOR, when cellular ATP levels are low, the activation of adenosine 5′-monophosphate (AMP)–activated protein kinase (AMPK) enhances the inhibitory function of the Tsc1/Tsc2 tumor suppressor proteins on Rheb, a small GTPase essential for mTOR function [30]. Consequently, decreased mTOR activity triggers autophagy, thereby ensuring that the cell adapts to its changing environment by slowing down growth and increasing catabolic processes.

Autophagy occurs constitutively in all eukaryotic cells and operates at fundamental levels, assuming a homoeostatic mechanism by regulating the degradation of molecules and the turnover of organelles [16]. In this context, autophagy is directed toward the degradation of misfolded protein cargos, thereby preventing the accumulation of the relevant proteins and consequent toxicity that may ultimately result in cellular damage and mortality [31]. Autophagy is rapidly induced under conditions of glucose or amino acid deprivation, oxidative stress, hypoxia, and exposure to xenobiotics, all of which may initiate or exacerbate cellular injuries [32]. Therefore, autophagy is not only a dynamic adaptation pathway but also safeguarding of proteome integrity and energy metabolism. Paradoxically, excessive autophagy has been observed in association with cell death; controlled autophagy is protective by providing essential substrates [33]. However, to aviod confusion, the term “autophagic cell death” has been restated as “cell death with autophagy” to describe cell death that is suppressed by inhibition of the autophagy pathway and led to a disruption in the autophagic flux [34]. Autophagic flux refers to the whole process of autophagy, and there are various methods to monitor autophagy [35]. An ideal method to assess autophagic activity is measuring the LC3-II levels, but it is crucial to complement this with an examination of substrate degradation (e.g., SQSTM1/p62) [35]. Furthermore, confirming changes in autophagic flux can be achieved through genetic modifications (like using short interfering RNA for ATG genes), using pharmaceutical inhibitors such as 3-methyladenine (3-MA) and chloroquine, or employing inducers like rapamycin [35].

### 2.2. The Role of Autophagy in IPF

IPF is a fatal chronic interstitial lung disease that impacts both lung mechanical functions and gas exchange. With the emergence of advanced molecular diagnostics, it is increasingly apparent that the pathogenesis of IPF is intricate, involving multiple molecular pathways, and thus is likely to necessitate diverse treatment strategies [6, 36].

Altered autophagy in fibroblasts has also been documented as a crucial factor in the pathogenesis of human IPF [37]. Notably, autophagic activity was abnormally low in IPF fibroblasts, which was attributed to the low expression of FoxO3a leading to a reduced level of LC3B transcription, ultimately causing a decreased autophagic flow in fibroblasts [38, 39]. Defective autophagy is necessary to maintain a cell death-resistant phenotype in fibroblasts within a collagen-rich matrix [20, 38]. The potential profibrotic function of autophagy in IPF fibroblasts necessitates a reevaluation of the utilization of autophagy activators in the treatment of IPF, with a focus on context-specific approaches.

**Figure 1:**
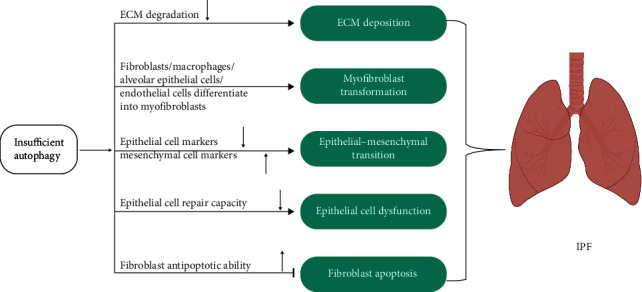
Autophagy process in the IPF. Altered autophagy promotes ECM production, myofibroblast transformation, epithelial–mesenchymal transition, epithelial cell dysfunction, and inhibits fibroblast apoptosis.

Autophagy is also involved in promoting profibrotic effects in IPF fibroblasts, so the utilization of autophagy activators for the treatment of IPF requires a context-specific approach. Recent evidence highlights the pivotal contributions of disrupted mitochondrial homeostasis in alveolar epithelial type II cells (AECIIs), fibroblasts, and alveolar macrophages (AMs) to the pathogenesis of IPF [40] (Figure 1). For instance, the accumulation of dysmorphic and dysfunctional mitochondria within AECIIs has been reported in the pulmonary of IPF patients [40]. The compromised mitochondria in AECIIs are linked to reduced PINK1 levels and impaired mitophagy. PINK1-deficient mice demonstrate disrupted mitochondrial homeostasis and the onset of PF [40]. The expression of PARK2, another protein associated with mitophagy, is decreased in the lung fibroblasts of IPF patients. PARK2 deficiency exacerbates bleomycin-induced PF in mice by enhancing myofibroblast differentiation and proliferation through the promotion of the PDGFR-PI3K-Akt signaling pathway [41]. Pirfenidone, an FDA-approved therapy and an exciting landmark in the field of IPF treatment, exerts its antifibrotic effects partially through the induction of PARK2-mediated mitophagy and the inhibition of myofibroblast differentiation [42]. Mitophagy, a subtype of macroautophagy, is elevated in profibrotic AMs [43]. During the fibrotic process, Akt1-mediated mitochondrial reactive oxygen species (ROS) induction triggers mitophagy in AMs, thereby influencing macrophage apoptosis resistance and the expression of TGF-*β*1 [43]. The TGF-*β*1 derived from AMs is required for PF, which promotes the differentiation of fibroblasts into myofibroblasts and the development of PF [43]. Furthermore, AECIIs treated with TGF-*β*1 were shown to induce mitophagy but TGF-*β*1 reduced mitophagy in fibroblasts by activating Akt in IPF lungs [43]. Considering the varying impact of mitophagy on different cell types in the development of IPF targeting cell type-specific mitophagy could lead to more effective therapeutic results in the treatment of IPF.

## 3. Role of Autophagy in the Therapeutic Potential of MSCs

**Figure 2:**
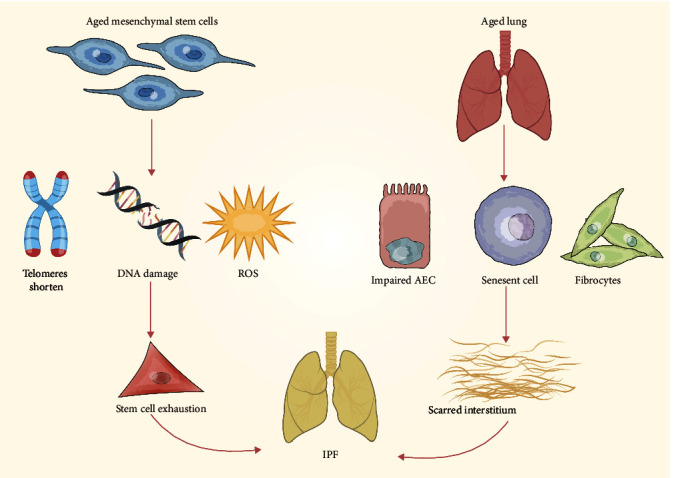
Aging characteristics of MSCs in IPF.

Since 1995, first tested MSCs have been gained wide popularity and extensively studied in preclinical model [44]. MSCs afford several advantages, such as easy accessibility, low immunogenicity, and therapeutic potential in regenerative medicine [45]. Due to these properties, MSCs have become very promising tool for therapy in different disease types and ideal cells in the treatment of IPF [46]. Initially, the beneficial effects of MSC-based therapies were attributed to the replacement capacity of MSCs [47]. However, this view has not stood the test of time; studies have revealed that structure and function of injured tissues by direct cell replacement are not the primary property of MSCs [48, 49]. Research to date have demonstrated that MSCs-derived secretome, which comprises a series of bioactive molecules and extracellular vesicles (EVs), plays a key role in immune modulation and promoting tissue repair [50, 51]. The keratinocyte growth factor (KGF), hepatocyte growth factor (HGF), and epidermal growth factor (EGF) derived from MSCs are helpful in tissue repair promoting effects. MSC-derived vascular endothelial growth factor (VEGF) has also been studied extensively for its angiogenic properties, which promote reepithelialization and angiogenesis [52]. MSCs reprogram proinflammatory macrophages (M1) toward an antiinflammatory phenotype (M2) resulting in exerting antifibrotic effects [53]. Furthermore, MSCs exert potent antifibrotic effects via modulating the ratio of metalloproteinases/metalloproteinase tissue inhibitors [54, 55]. Given that IPF is an age-related disease, recent studies have found that MSCs exhibit aging under sustained pathological conditions such as chronic injury and oxidative stress, which affects the therapeutic activity of MSCs and leads to PF [56] (Figure 2).

Recently, it has been proposed that autophagy in MSCs is potentially a new approach for improving therapeutic effects of MSCs (Table 1). Autophagy plays a dual role in MSCs: (1) Modulating autophagy in MSCs may control the proliferation, activation, and effector function of MSC; (2) MSCs are able to modulate the autophagy of immune and other cells that play an important role in the pathogenesis of inflammatory lung diseases [67]. Both of these mechanisms eventually affect the efficency of MSC-based therapy. The initial observation indicating the crucial involvement of autophagy in MSC processes was the disparity in autophagosome quantities between undifferentiated MSCs and their differentiated counterparts [68]. Furthermore, the hindrance in the fusion between autophagosomes and lysosomes, resulting in the obstruction of autophagosome degradation, culminates in the accumulation of autophagosomes within undifferentiated MSCs [69].

A recent study indicates that inhibiting autophagy enhances the immune-suppressing abilities of MSCs [70]. The research reveals that reducing the expression of Becn1 gene in MSCs (short hairpin Becn1-MSCs) strengthens their therapeutic and immune-modulating effects [70]. Notably, when treated with these modified short hairpin Becn1-MSCs, a more pronounced decrease in the populations of CD4+ and CD8+ T cells, as well as a reduced proliferation of MOG (myelin oligodendrocyte glycoprotein)-specific CD4+ T cells, is observed, all without impacting the polarization of T cells [70]. Similar results were achieved when these mice received MSCs that had been pretreated with an autophagy inhibitor [70].

The modulation of MSC autophagy can significantly influence their secretion capacity, thereby impacting their overall functionality [71]. Notably, when MSCs are pretreated with the autophagy-inducer rapamycin and subsequently subcutaneously injected, it results in an augmentation of their wound-healing potential. This enhancement is closely linked to the promotion of angiogenesis, driven by the autophagy-induced secretion of VEGF [71]. Conversely, MSCs in which BECN1 is silenced, causing an early blockade of the autophagic machinery, exhibit a diminished therapeutic effect [71].

Thus, modulation of autophagy in MSCs seems to be a potential target to enhance the therapeutic properties of MSC-based therapy, but great action needs to be taken, and further studies should be conducted.

## 4. Importance of Autophagy in Maintaining Healthy MSCs

### 4.1. Excessive Autophagy Promotes Apoptosis of MSCs

**Figure 3:**
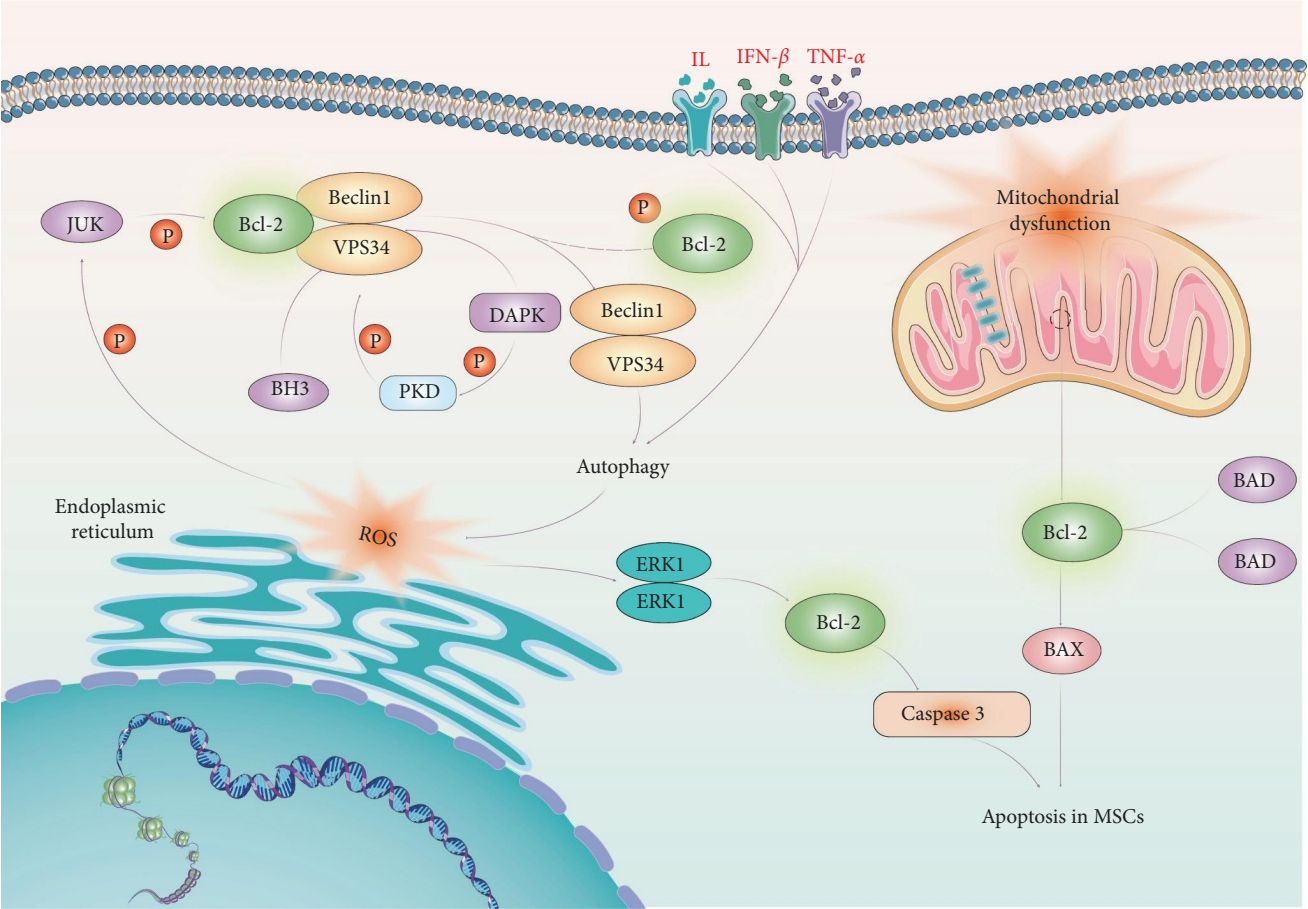
Excessive autophagic activity promotes apoptosis of MSCs under inflammatory microenvironment.

MSCs are a heterogeneous population of multipotent stromal stem cells that can be easily isolated from a variety of different sources [72]. MSCs offer diverse benefits that stem from their and the ability to differentiation into osteoblasts, chondrocytes, and adipocytes under appropriate and specific stimuli [73, 74]. Additionlly, MSCs exert an immunomodulatory effect on innate and adaptive immune responses via interaction with the inflammatory microenvironment [75, 76]. Therefore, MSCs have been widely used in clinical trials to treat autoimmune and inflammatory diseases, particularly in the context of lung injuries [77]. However, there is a lack of comprehensive understanding regarding the precise impact of the inflammatory microenvironment on the fate of MSCs. The inflammatory microenvironment plays a key role in mediating immunoregulatory capability of MSCs [76, 78]. MSCs exert enhanced immunosuppressive functions after interaction with inflammatory cytokines, including interferon (IFN)-*γ*, tumor necrosis factor (TNF)-*α*, interleukin (IL)-1*α*, and IL-*β* [74, 79] (Figure 3). Related literature has shown that both fetal and adult MSCs are susceptible to lysis by IL2-activated natural killer cells [80]. Furthermore, IFN-*γ* synergistically amplifies TNF*α*-induced apoptosis in MSCs, thus impeding their capacity to repair damaged lung tissue, indicating that apoptosis of MSCs could be induced in the inflammatory microenvironment during the development of PF [81].

Recent research has demonstrated that TNF-*α* and IFN-*γ* inflammatory cytokines such as IFN-*γ* and TNF-*α* activate autophagy in MSCs by upregulating Beclin 1 expression, which attenuates the immunosuppressive capacity of MSCs [19]. Although autophagy has been considered a cell survival mechanism, it can also promote cell death depending on the specific physiological and pathological conditions; the dual function of autophagy in prosurvival and prodeath remains incomplete [82, 83]. Autophagy constitutes major adaptive (survival) strategy of cells in response to challenges such as starvation, growth factor withdrawal, and neurodegeneration but is also a critical contributor to the death of certain types of cells [84, 85]. There is evidence to support autophagy promoted TNF-*α* plus IFN-*γ*-induced apoptosis of MSCs, highlighting the varied functions of autophagy under conditions of inflammation and nutrient scarcity [19]. Consequently, it is feasible to consider the manipulation of autophagy in MSCs as a means to optimize therapeutic effectiveness.

### 4.2. Impact of Declined Autophagy on MSCs Aging

As MSC populations with systematic age, they undergo functional deterioration and less effective in vivo or extended culture in vitro, limiting their therapeutic applications [86–88]. The underlying processes that drive MSCs senescence remain unclear, but significant progress has been made in elucidating the aspects of age-related MSCs phenotypic changes as well as possible mechanisms that influence MSCs senescence [89].

Autophagic activity tends to decrease with age across various model organisms, potentially leading to the buildup of autophagic structures and constraining the capacity for maintaining cellular homeostasis in certain contexts [90–92]. Human cell studies have revealed that age-related declines in the breakdown of lysosomal proteins hinder the autophagic flux, worsening cellular damage and playing a role in the onset of age-related diseases [93–96]. Additional evidence has substantiated that aging is linked to a diminished expression of several Atg genes, including Atg2 and Atg8a, which play a crucial role in both the initiation and functionality of autophagy [97]. In normally aged mice, autophagy was significantly reduced, as indicated by decreased levels of Atg7, LC3-II, autophagosome, autophagolysosomal fusion, autophagy substrates, and autophagy receptor [98]. Consistent with this, autophagy was attenuated in both aged rat brain tissue and aged human fibroblasts, as evidenced by significantly decreased levels of autophagy-associated proteins, such as Atg5-Atg12 and Becn1, and significantly increased levels of mTOR and ferritin H [99]. In normal older human brain samples, the expression of key autophagy genes like Atg5 and Atg7 was also reduced [100]. Additionally, several age-related human pathologies are closely linked to deficits in autophagy that develop and progress with age [101–103]. Taken together, compromised autophagy is a characteristic of organismal aging, as autophagy abundance declines with age and cargo is not delivered to the lysosomes as efficiently.

On the contrary, research on long-lived mutant animals has revealed that increased autophagy is linked to delayed aging. Specifically, the prolonged lifespan observed in *C. elegans* daf-2 loss of function mutants relies on autophagic genes like bec-1, lgg-1, atg-7, and atg-12 [92, 104, 105]. Moreover, the extended longevity in various longevity mutants, including daf-2 mutants with reduced insulin/insulin-like signaling, germline-less glp-1(e2141) mutants, dietary-restricted eat-2 (ad1116) mutants, mitochondrial respiration-defective clk-1(e2519) mutants, and mRNA translation-impaired rsks-1 (sv31) mutants, necessitates the presence of HLH-30 [106]. Activation of autophagy with rapamycin could restore the proliferative function of aged MSCs [107]. These findings align with evidence of reduced induction in autophagosome formation and lysosomal degradation in the absence of HLH-30, suggesting that HLH-30 plays a pivotal role in promoting longevity by regulating the autophagic process downstream of various lifespan-extending mechanisms [106]. Further, the formation of long-lived dauer worms, which correspond to a larval hibernation stage, is correlated with increased autophagy and depends on autophagy genes atg-1, atg-7, lgg-1, and atg-18, demonstrating the importance of autophagy to organismal adaptation in challenging conditions [105]. However, impaired autophagy could increase ROS and lead to MSC aging [108]. Similarly, high glycemic treatment of MSCs increased ROS-mediated autophagy, leading to the formation of Beclin-1, Atg5, Atg7, Atg12, and LC3-II autophagosomes, which induced MSC aging and local inflammation [109].

**Figure 4:**
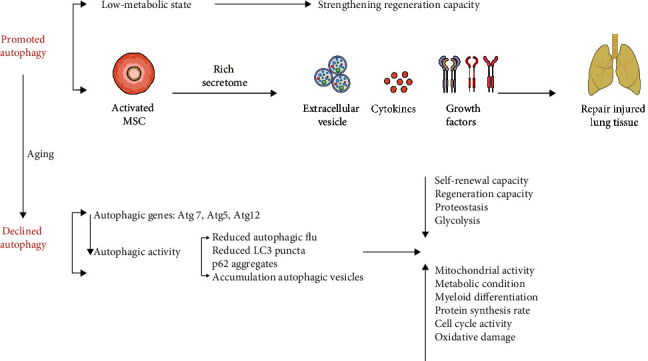
Autophagy influences MSCs activity and aging. In MSCs, promoted autophagy partially reverses the aging of MSCs, while declined autophagy attenuates the biological functions of MSCs.

Together, collective research suggested that (1) autophagy is impaired during as MSCs undergo aging, (2) autophagy dysfunction shortens the lifespan of MSCs, and (3) enhancing or restoring autophagy prolongs the lifespan and extends the healthspan of MSCs (Figure 4). This demonstrates that autophagy regulation is central to the aging of MSCs (Table 2).

## 5. Targeting Autophagy in IPF

Treatment choices for IPF are quite restricted. While recent trials have demonstrated the effectiveness of pirfenidone and nintedanib in slowing the decline of lung function in IPF patients, no medication can reverse or entirely prevent the progression of IPF [110, 111]. IPF has emerged as the most prevalent indication for lung transplantation, with a 5-year survival rate posttransplant just slightly exceeding 50% according to the International Society of Heart and Lung Transplant (ISHLT) registry [112, 113]. However, lung transplants continue to face significant clinical constraints, primarily due to the shortage of available donors [114]. In addition to investigating autophagy mechanisms in IPF, multiple drugs have been introduced to mitigate the progression of the disease [115]. Furthermore, an array of compounds with therapeutic potential in IPF by modulating autophagy are steadily emerging [116, 117].

## 6. Current Drugs to Treat IPF

In the past 10 years, researchers have spent a lot of effort on IPF drug design, but still only two approved drugs, pirfenidone (Pirfenidone) and nintedanib (NIT), have been used in patients with IPF. Pirfenidone and nintedanib have yielded a discernible elevation in mortality and PF progression among IPF patients under clinical observation [118]. Pirfenidone exerts its antifibrotic effects primarily through inhibition of TGF-*β*1, a critical mediator involved in IPF development [119, 120]. Pirfenidone, an oral pyridine, reduces extracellular matrix (ECM) deposition via interfering with collagen production and fibrinolytic processes by reducing the production of certain tissue necrosis factors and growth factors [121–123]. Notably, pirfenidone can activate ATG7- and ATG5-dependent canonical autophagy in lung fibroblasts, as a decrease in EGFP-LC3 dot formation as well as LC3 conversion from LC3-I to LC3-II was observed when ATG5 and ATG7 were knocked down [42]. Although pirfenidone induced autophagy has been clearly demonstrated, the precise mechanism of pirfenidone inhibiting lung fibrosis via autophagy during IPF pathogenesis should be futher examined.

Nintedanib is another therapeutic medication possessing antifibrotic attributes, operating as a multityrosine kinase inhibitor (MTKI) [124]. Nintedanib can inhibit the fibrosis process by targeting PDGFR*α*-*β*, FGFR1-3, VEGFR1-3, and SFK [125–129]. Nitidanib has shown antifibrotic and anti-inflammatory activity in animal models of lung fibrosis, interfering with fibrotic processes such as fibroblast proliferation, migration, and differentiation and significantly reducing the deposition of lung collagen [130, 131]. In addition, efficacy and safety of nintedanib in patients with IPF have been demonstrated in phase 3 clinical trials, reducing the decline in forced vital capacity (FVC) and slowing the progression of fibrosis [120]. Furthermore, certain studies have substantiated the ability of nintedanib to restrain the growth of specific lung vascular cells, including endothelial cells and pulmonary artery vascular smooth muscle cells [131]. Notably, the research revealed that nintedanib effectively boosted autophagy by assessing the LC3-I/II ratio [132]. Another investigation produced consistent findings, confirming that nintedanib enhanced autophagic flux in fibroblasts confirmed by observing increased LC3-II formation and induced Beclin-1-dependent, ATG7-independent autophagy in fibroblasts [133]. Presently, due to extensive research into autophagy regulation, several autophagy-targeted pulmonary antifibrotic treatments have been identified [134, 135].

## 7. Potential Compounds to IPF

Amounting research mainly to identify the new molecular targets and therapy choices. Berberine, an important protoberberine alkaloid, shows various pharmacological activities that have been widely used in different therapeutic areas [136]. Berberine is extensively distributed in a variety of herbs and its synthetic derivatives have gained significant interest in clinical applications [136]. Importantly, berberine as an autophagy modulator can be efficient against PF via modulating autophagy [137, 138]. Berberine can remarkably enhance the expression of LC3 and Beclin-1, while significant attenuation of p-mTOR, Akt, and MAPK signaling pathways, thereby stimulating autophagosome formation and initiating autophagy [139, 140].

Spermidine, an autophagy-inducer, enhances Beclin-1-dependent autophagy and autophagy modulators in IPF fibroblasts and bleomycin-induced mouse lungs [141]. Specifically, spermidine upregulated autophagic flux, leading to an increase in the LC3B-I/II ratio and the expression of ATG7 and Beclin-1 in IPF fibroblasts and bleomycin-induced mouse lungs [141]. In addition, spermidine can reverse autophagy impairment by decreasing the expression of p-mTOR in bleomycin-induced lungs [141]. These finding demonstrate that spermidine enhances autophagy and that this effect may hold promise in the treatment of IPF.

Immune checkpoint PD-1 play a critical role in controlling inflammatory response to injury in the normal lung tissues. Programed death ligand-1/programmed cell death 1 (PD-L1/PD-1) axis is one of the most essential immune checkpoints in regulating immunotherapy. In IPF patients, PD-L1 was found to have overexpression on alveolar macrophages (AMs) but was negative on fibroblasts and myofibroblast membranes [142, 143]. Blocking PD-L1 can reverse PF by increasing phagocytosis of profibrotic fibroblasts in vivo mouse model of fibrosis [144]. The anti-PD-L1 monoclonal antibody (anti-PD-L1 mAb) has been discovered to significantly inhibit the proliferation and migration of lung fibroblasts and reduce the deposition of ECM [145]. It can increase the expression of the autophagy-related marker protein SQSTM1 and the accumulation of LC3II, promote the formation of autophagosomes, and ultimately induce autophagy activation in PF [145]. These evidences show that anti-PD-L1 therapy has the potential to alleviate PF, offering a novel approach to treating IPF.

Bergenin, a compound derived from a variety of medicinal plants, is a major component from *Bergenia stracheyi* (Saxifragaceae) [146]. Bergenin could attenuate bleomycin-induced PF in mice by suppressing the myofibroblast activation and promoting the autophagy and the apoptosis of myofibroblasts [147]. The study revealed that berberine significantly reduced the phosphorylation levels of mTOR, ULK1, and S6 and decreased the expression levels of typical fibroblast activation markers *α*-SMA and ECM protein collagen I, thus promoting autophagy and alleviating PF [147]. Moreover, bergenin has the potential to maintain normal autophagy and apoptosis balance in IPF fibroblasts by modulating energy metabolism [147]. Overall, there is a pressing requirement for additional investigations and animal model assessments to facilitate the development and validation of novel therapeutic agents for IPF that specifically target autophagy.

## 8. Conclusion

With the developments in regenerative medicine technology, stem cell therapy has been tested for safety and efficacy in various lung diseases. However, the abnormal health status of MSCs can affect their own therapeutic function, especially in IPF. The new evidence indicates that modulation of autophagy in MSCs plays an important role in the therapeutic action exerted by MSCs. To either induce or inhibit autophagy activity in lung tissue microenvironment can affect the ability of MSCs to repair damaged tissues, specially IPF. Elevating autophagy generally enhances cellular functions and maintains homeostasis, contributing to prolonged lifespan and improved pulmonary health. However, it is crucial to recognize that a substantial increase in autophagy may potentially reduce lifespan and adversely affect lung health. The therapeutic targeting of autophagy in aging and age-related lung diseases is contingent upon the specific autophagic defects present in different cell types. From existing literature, it can be postulated that enhancing autophagic activity to augment MSCs function in IPF represents a promising therapeutic strategy to enhance lung function in the elderly. The sustained health benefits for MSCs are likely to result from achieving an optimal balance of autophagy and are influenced by both lung tissue and organismal age. This review aims to provide more comprehensive insights into how autophagy affects the therapeutic properties of MSCs, thereby broadening the horizon of clinical utilization of MSCs for the treatment of IPF. The development of novel MSC therapies targeting the autophagy signaling pathway may provide an innovative and attractive approach to the field of regenerative medicine.

## Figures and Tables

**Table 1:** Role of autophagy in the therapeutic potential of MSCs.

Disease model	Mechanism	Autophagy effect	Reference
Ischemic stroke	MSCs inhibit autophagy and promote cell survival by transferring miR-25 to support recovery of neurological function after stroke	Negative	[57]
Liver fibrosis	Autophagy inhibition via Becn1 downregulation improves the MSCs antifibrotic potential	Negative	[58]
Hypoxic-ischemic brain damage	MSCs reduce autophagy in hippocampal neurons partly through the AMPK/mTOR pathway	Negative	[59]
Osteoarthritis	MSCs enhance autophagy in chondrocytes via mTOR inhibition and protect articular cartilage from damage	Positive	[60]
Acute lung injury	MSCs enhance autophagy and ameliorate acute lung injury partially via delivery of miR-100	Positive	[61]
Idiopathic pulmonary fibrosis	Inhibition of miR-199a-5p enhances autophagy by regulating the Sirt1/AMPK signaling pathway and rejuvenates IPF-MSCs senescence	Positive	[62]
Parkinson's disease	MSCs enhance autophagy and exert a neuroprotective effect through the modulation of *α*-synuclein	Positive	[63]
Alzheimer's disease	MSCs enhance autophagy and increase *β*-amyloid clearance to improve neuronal survival against A*β* toxicity	Positive	[64]
Inflammatory bowel disease	Enhancement of autophagy in MSCs improves immunosuppression of MSCs by increasing Pacer levels	Positive	[65]
Diabetic kidney disease	MSCs diminish cell death in kidney tissue facing diabetic kidney disease, culminating in podocyte maintenance, and also downregulating the over induction of the autophagy pathway	A double-edged sword	[66]

**Table 2:** Autophagy modulation on apoptosis and aging in MSCs.

Experimental model	Molecular mechanisms	Autophagy effect on MSCs	References
Cecal ligation and puncture mouse model	Inflammatory microenvironment-induced autophagy inhibits the expression of the prosurvival gene Bcl-2 via suppressing reactive oxygen species/mitogen-activated protein kinase 1/3 pathway	Promotes apoptosis	[19]
Mice model	Activation of autophagy could reduce the adipogenic differentiation and promote proliferation of aged MSCs	Reverses aging	[107]
Mice model	Inhibition of autophagy could turn young MSCs into a relatively aged state by reducing their osteogenic differentiation and proliferation capacity and enhancing their adipogenic differentiation capacity	Promotes aging	[107]
Mice model	Impaired autophagy led to increased ROS and further induced the p16INK4a axis	Promotes aging	[108]
Cellular experiment	High glycemic treatment of MSCs increased ROS-mediated autophagy, leading to the formation of Beclin-1, Atg5, Atg7, Atg12, and LC3-II autophagosomes, which induced MSCs aging and local inflammation	Promotes aging	[109]

## Abbreviations

IPF: Idiopathic pulmonary fibrosis
MSCs: Mesenchymal stem cells
AECs: Alveolar epithelial cells
PF: Pulmonary fibrosis
MA: Macroautophagy
AMP: Adenosine 5′-monophosphate
AMPK: AMP-activated protein kinase
3-MA: 3-methyladenine
AECIIs: Alveolar epithelial type II cells
AMs: Alveolar macrophages
ROS: reactive oxygen species
EVs: Extracellular vesicles
KGF: Keratinocyte growth factor
HGF: Hepatocyte growth factor
EGF: Epidermal growth factor
VEGF: Vascular endothelial growth factor
IFN: Interferon
IL: Interleukin
ISHLT: International society of heart and lung transplant
NIT: Nintedanib
ECM: Extracellular matrix
FVC: Forced vital capacity
PD-1: Programed death-1
PD-L1: Programed cell death ligand-1
AMs: Alveolar macrophages
Anti-PD-L1 mAb: anti-PD-L1 monoclonal antibody.
